# Infrared Spectroscopy for Online Measurement of Tars, Water, and Permanent Gases in Biomass Gasification

**DOI:** 10.1177/0003702821991891

**Published:** 2021-02-18

**Authors:** Mohit Pushp, Christian Brackmann, Kent Davidsson

**Affiliations:** 1 Research Institutes of Sweden, Borås, Sweden; 2 Department of Chemistry and Molecular Biology, 3570 University of Gothenburg, Gothenburg, Sweden; 3 Division of Combustion Physics, Lund University, Lund, Sweden

**Keywords:** On-line tars, gasification, hydrocarbons, infrared spectroscopy

## Abstract

Online measurements of the raw gas composition, including tars and water, during biomass gasification provide valuable information in fundamental investigations and for process control. Mainly consisting of hydrocarbons, tars can, in principle, be measured using Fourier transform infrared (FT-IR) spectroscopy. However, an instrument subjected to raw gas runs the risk of condensation of tars on optical components and subsequent malfunction. Therefore, an external cell, heated to at least  400 ℃, has been designed to ensure that tars remain in the gas phase during FT-IR measurements. The cell was used for on-line FT-IR measurements of permanent gases (CO, CO_2_, CH_4_), water, and tars during the operation of a lab-scale downdraft gasifier using wood pellets, bark pellets, and char chips. Based on calibration, the measurement error of permanent gases was estimated to be 0.2%. Concentrations evaluated from spectral signatures of hydrocarbons in tar are in good agreement with results from solid-phase adsorption measurements and correlated well with operational changes in the gasifier.

## Graphical abstract

**Graphical Abstract:**
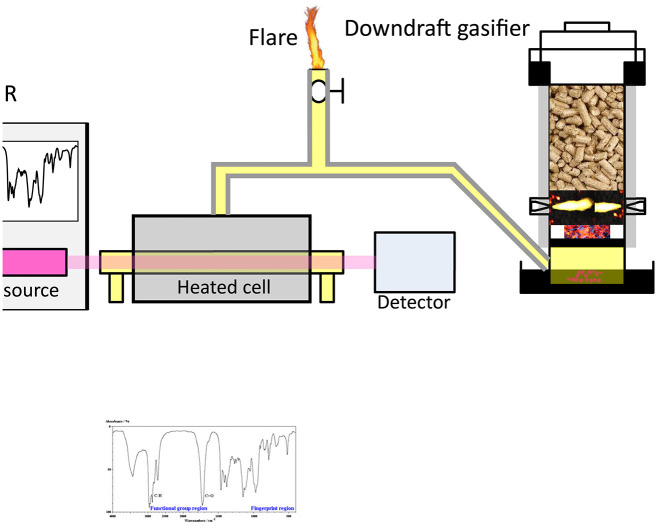

## Introduction

Gasification processes can provide efficient and economical use of biomass as the product gas can be upgraded to high-value end products like synthetic natural gas, fuels for heavy vehicles, chemicals, etc. One of the challenges of biomass gasification is the relatively large fraction of tars, i.e. larger hydrocarbons, released during the devolatilization.^1−4^ In comparison with steady-state operation, the tar content is significantly higher during the start-up of the gasifier, addition of fresh fuel, etc.^5^ The fraction of tars can be minimized by controlling the gasification process or by physical separation in downstream components. Cracking of tars thus occurs both thermally and catalytically in a gasification unit. Methods for tar analysis, such as solid-phase adsorption (SPA) and physical adsorption of tars in organic solvents, are commonly practiced in the field, and detailed information about the species can thus be obtained. However, both are off-line techniques that lack temporal resolution, average out peak values, and require at least a couple of hours to obtain results.^6–8^ The residence time of the tar-laden gas at high temperature and operational conditions change the composition of tar compounds quite rapidly.^8^ Therefore, rapid information about tar concentrations is a prerequisite for improved process control, especially during start-up or while changing operational conditions.

Online techniques for tar measurement include flame ionization detection (FID), photoionization detection (PID), and spectroscopic methods.^9−11^ Analysis with FID is based on a comparison between the response of gas with and without tars. The method has some drawbacks; for instance, it is difficult to account for the decrease in gas volume because of water condensation while removing tars. Furthermore, regular calibration is required as the values are very sensitive to parameters like carrier gas volume and pressure. Results are also dependent on the accuracy of tar removal, especially at lower concentrations. In PID, photons ionize hydrocarbon molecules, and their movement toward a negatively charged electrode results in a current proportional to the concentration of the compounds. The ionization potential of higher molecular weight hydrocarbons like pyrene, biphenyl, naphthalene, cresol, phenol, and toluene lies in the range of 7.5–9 eV. Signatures of tars can thus be obtained using a suitable UV light source, and the sensitivity of the PID method is about 50 times higher than that of FID. However, the quantification is dependent upon the presence of molecules with low ionization potential and the stability of the tar compounds in the gas stream.^10^

Spectroscopic techniques for online measurements of tars include laser-induced fluorescence (LIF) based on the absorption of light by a molecule followed by a short time delay during which the molecule has extra energy and resides in an excited state before it emits light (fluorescence). Tar measurements with LIF in gasification were made using powerful ultraviolet lasers as well as compact, low-power, light-emitting diodes.^11^ While the LIF technique offers sensitive detection of low concentration levels, the signal is difficult to convert into quantitative concentrations. The signal has limited specificity for individual tar compounds as fluorescence spectra are broad and very similar for many hydrocarbons. Raman spectroscopy, based on the scattering of light, measures hydrocarbons by their molecular vibrations and can give spectra with better specificity.^12^ The Raman signals are, however, weak, and to measure online Raman spectra in hot gas requires a high-power laser. While these types of measurements are feasible in small laboratory setups, it would be more challenging in large-scale gasifiers with restricted optical access and higher concentrations of particles in the measurement volume.

Molecular vibrations are also probed through absorption in the infrared wavelength regime, which is the basis of Fourier transform infrared (FT-IR) spectroscopy, a well-established method for analysis of gas composition with high sensitivity.^13^ Tars absorb in the infrared wavelength regime and can, in principle, be measured by FT-IR. Even though limitations exist because of gas sampling and condensation, the FT-IR technique has been applied in fundamental studies of pyrolysis and gasification in laboratory-scale experiments with rather small amounts of fuel, often in combination with thermo-gravimetrical analysis.^14,15^ Examples of such studies are measurements of biomass components (cellulose, hemicellulose, and lignin) by Liu et al.^14^ and Biagini et al.,^15^ detection of formaldehyde–CO–CO_2_ by Li et al.,^16^ and of nitrogen compounds HCN–NH_3_–HNCO by Ren et al.^17^ The FT-IR technique is also well established for studies of combustion, and instruments for measurements in industrial environments are available.^18^

Commercial FT-IR instruments typically have a built-in gas cell with mirrors arranged for multiple passages of the infrared light. The cell is heated to 175–200 ℃ to avoid condensation of, e.g., water. Tars, however, may condense in the range 200–350 ℃, and therefore measurements during gasification using such a device would not be feasible because of tar condensation and subsequent damage to the optical components in the built-in cell.

A specially designed cell for FT-IR analysis at high temperatures has been presented previously for spectroscopic studies of CO_2_.^19^ Thus, the FT-IR technique has the potential to be applied in gasification if the infrared light can be guided through a sufficiently heated cell. The results can then provide valuable input to the process control. With this as the overall objective, the present study aimed to investigate if an external heated cell attached to an existing FT-IR spectrometer can provide reliable online concentration measurements of permanent gases like CO, CO_2_, and CH_4_ as well as of tars and water. An additional aim was to investigate if the online measured species concentrations could be related to the operational changes in the gasifier.

## Experimental

### FT-IR, Heated Cell, and Gasifier Setup

**Figure 1.**
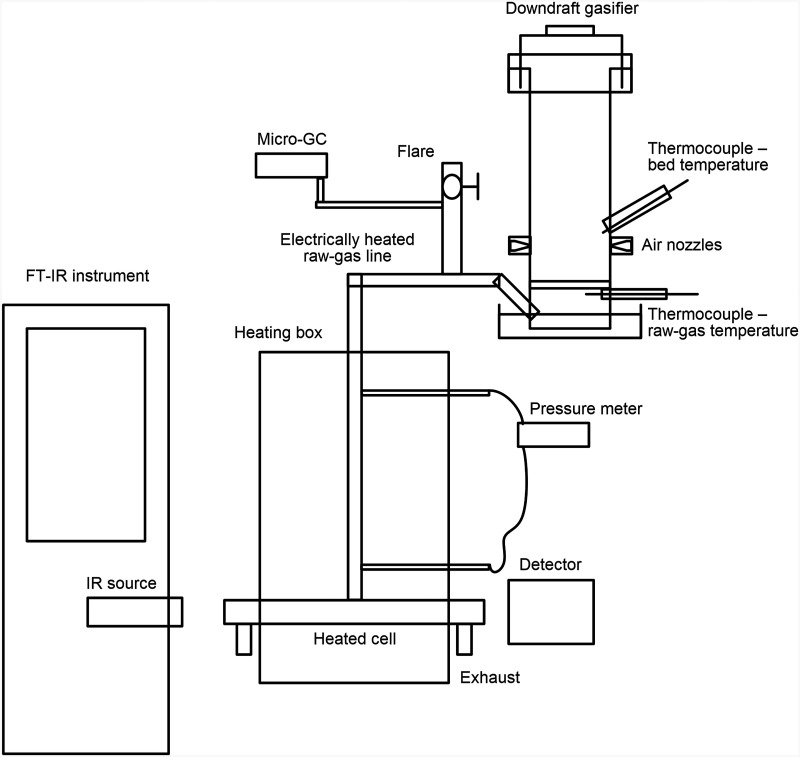
Schematic of the experimental setup of the FT-IR spectrometer connected to an external heated cell and a bench-scale gasifier. Part of the generated gas is led to the external measurement cell using a heated tube for FT-IR analysis while the remaining gas is led to a burner for flaring. A gas chromatograph is connected to the system for comparative measurements of concentrations. FT-IR: Fourier transform infrared; GC: gas chromatography.

A schematic of the experimental setup, including a downdraft gasifier and an FT-IR instrument with an external heated cell, is shown in Fig. 1.

The FT-IR spectrometer (Bomem model MB100) allows for absorption measurements in the infrared regime for wavenumbers 750–4000 cm^−1^ at a spectral resolution of 1 cm^−1^. The spectrometer is equipped with a gas cell with mirrors arranged for a total absorption path length of 6 m and also has a side port through which the IR radiation could be guided. This port allows the detector to be placed for measurements outside the standard cell. The possibility for the arrangement of an external cell is not specific to this model as the external port option is commonly provided in commercially available FT-IR instruments. An external cell fabricated using a stainless-steel tube, with an inner diameter of 26 mm and a length of 40 cm, was positioned between the IR source and the detector. The external cell and connecting tubes to the gasifier were enclosed in a heated box maintained at a temperature of at least 400 ℃ to avoid condensation of tars. The cell and the detector were aligned to obtain maximum transmission of light from the IR source. Nitrogen was used to purge the cell to obtain a reference background spectrum without absorption.

To investigate the concept of online FT-IR measurements during gasification, the heated cell was coupled to a specially fabricated bench-fed downdraft gasifier with average fuel consumption of 250 g/h. The gasifier was operated using wood, char chips, and bark pellets. The operation was disturbed intermittently by opening the top lid and stirring the bed manually, thereby affecting the porosity of the bed. The oxidation zone temperature was varied by adjusting the airflow for the gasification and measured by a thermocouple positioned at the center of the bed. Experiments were carried out in two steps: First, as a proof of concept, measurements were conducted using an open-ended cell, and second, measurements were made with the ends sealed with KBr windows. To maintain a constant flow of raw gases to the open-ended cell, the static pressure was measured between two points and adjusted manually during the measurements. This was also necessary to minimize variations due to dilution from ambient air while using an open-ended cell; however, with closed ends, such dilution was avoided. Results presented include data obtained both using open-ended and closed cell. The excess stream of gases from the gasifier was flared during gasifier operation, and the fact that the generated gas is flammable is an indication of its quality. An IR instrument (Rosemount model BINOS 100) was used to measure CO_2_ and CH_4_ in the raw gas, while O_2_ was measured using an oxygen analyzer (M&C PMA 10). The raw gas was then cooled down to 20 ℃ and passed through the particle filter. The concentrations of permanent gases were also analyzed using gas chromatography (GC) (Agilent 490 Micro-Gas Chromatograph). No oxygen leakage was observed within the extraction setup during GC measurements.

### Solid-Phase Adsorption Sampling in the Product Gas

A tar sampling port, electrically heated to at least 400 ℃, was situated downstream of the gasifier. Tars were collected on dual-layer SPA columns using Supelclean ENVI-Carb/NH_2_ SPE Tube (Sigma-Aldrich). The method was originally developed by Brage et al.^6^ A gas chromatograph coupled with a flame ionization detector (FID) was used to analyze different species of organic hydrocarbons in the SPA samples. The lab-scale gasifier was operated in separate events using wood pellets (0–120 min), bark pellets (120–240 min), and char chips (240–400 min). Operating conditions for the gasifier, such as airflow and bed temperatures, were maintained similarly to those employed for experiments with FT-IR measurements.

### FT-IR Spectral Analysis

According to the Beer–Lambert absorption law, the transmission of light, *T*, through the heated cell is given by Eq. 1

(1) $$T=\frac{I}{I_{0}}=e^{-N\sigma x}=e^{-\mathrm{kx}}$$

The absorbance is defined as the logarithm of the transmission, according to Eq. 2

(2) $$A=-\text{log}\frac{I}{I_{0}}=N\sigma x=\mathrm{kx}$$

In Eqs. 1 and 2, *I* is the transmitted intensity, while *I_0_* is the incident intensity measured when purging the system with nitrogen. *N* is the number density of the absorbing species, σ is the absorption cross-section, and *x* the absorption path length. Alternatively, the Beer–Lambert law can be written with an absorption coefficient *k*, often expressed in cm^−1^, as shown on the right-hand side of Eq. 1. The latter quantity for absorption is used in the HITRAN database employed for rendering theoretical spectra of gases CO, CO_2_, H_2_O, and CH_4_, used for data analysis.^20,21^ Absorption data for tar compounds can be found in literature, for example, from the United States Environmental Protection Agency (EPA) spectral database.^22^ Nevertheless, reference spectra for analysis of gasifier data were obtained from calibration measurements carried out on aromatic species, which are major tar components, including pyrene, anthracene, naphthalene, xylene, toluene, and benzene. A specific mass of a tar compound was vaporized using heated nitrogen maintained close to the tar boiling point in an additional stainless-steel tube (diameter = 12 mm) connected to the FT-IR cell. The tar vapor was transported into the FT-IR cell, maintained at temperature 400 ℃, by a flow of the nitrogen, and measurements were made continuously during the evaporation.

A transmission spectrum could be calculated according to Eq. 1 using the absorption coefficient for a species and the length of the cell. The transmission spectrum was convoluted with a Gaussian line-shape having a full width at half-maximum value of 0.83 cm^−1^ to account for the resolution of the FT-IR instrument. An absorbance spectrum was then calculated from the broadened transmission spectrum using Eq. 2 and could then, in turn, be compared with experimental data. The wavenumber range for evaluation of each species was selected to have absorbance values typically below 0.3 to ensure the applicability of the Beer–Lambert law.

Concentrations were evaluated by a least squares fit of experimental to calculated spectra compiled with contributions from CO, CO_2_, H_2_O, CH_4_, and tars. Sum of squares (SSQ) between calculated and experimental absorbance values for data points *j*, *A_sim,j_*, and *A_exp,j_* were calculated for wavenumber intervals corresponding to the spectral signatures of the different species according to Eq. 3

(3) $$\mathrm{SSQ}=\sum_{j}(A_{\mathrm{sim},j}-A_{\text{exp},j})2$$

The concentrations of the experimental spectrum were evaluated by the minimization of the SSQ parameter.

## Results

### Calibration of the External Cell Using Model Tar Compounds

**Figure 2.**
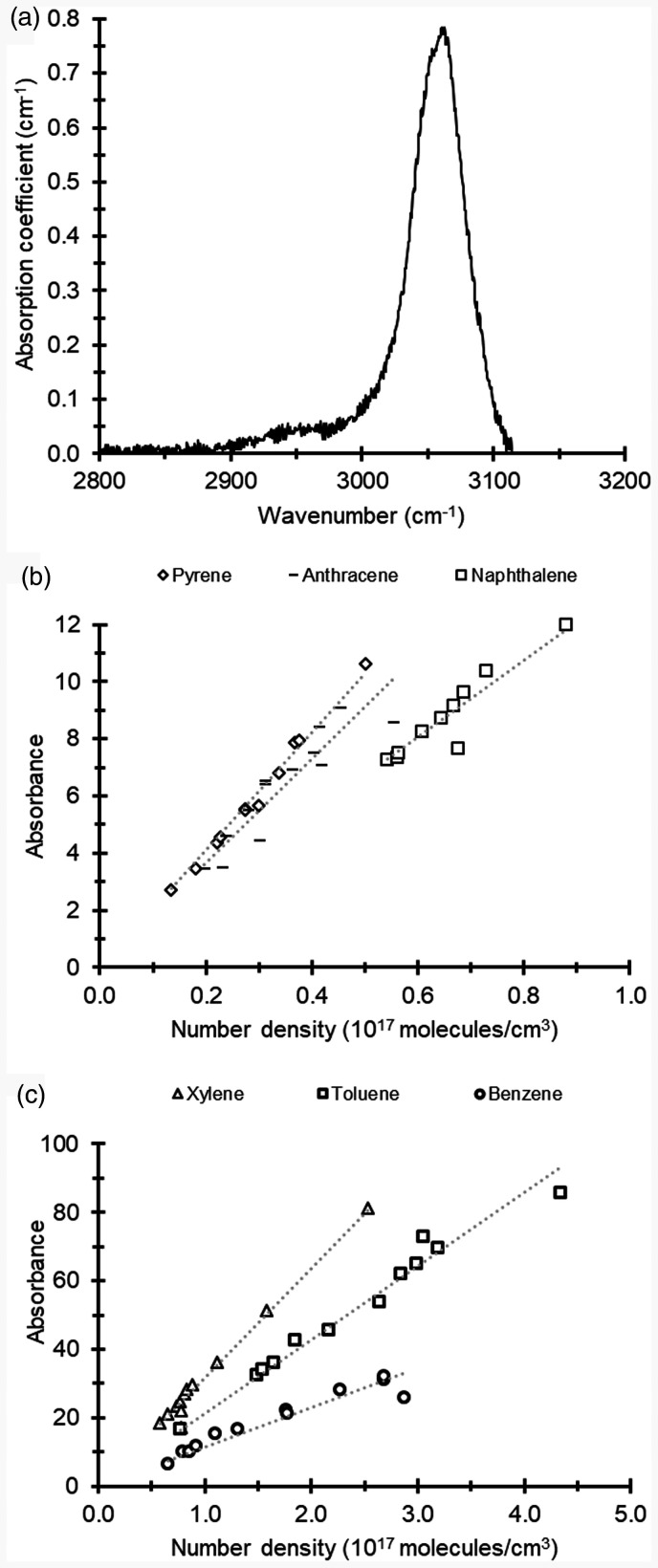
(a) FT-IR spectrum of naphthalene measured at 400 ℃ in the heated cell. Calibration curves of absorbance versus concentration for model tar compounds. (b) Pyrene, anthracene, and naphthalene. (c) xylene, toluene, and benzene.

Figure 2a shows an FT-IR spectrum obtained for naphthalene with a peak that represents stretching vibrations of the aromatic ring structure located at wavenumbers 3000–3100 cm^−1^.^23^ The absorbance integrated over wavenumbers 3020–3100 cm^−1^ was related to the tar compound concentration, and plots of absorbance versus number density are shown in Figs. 2b and 2c.

The data are presented together with linear fits, and since the absorption path length in the cell is the same for all measurements, the slope of a line represents the absorption cross-section (cf. Eq. 2). For comparison with literature data, the following absorption cross-sections integrated over the analyzed wavenumbers have been calculated; 1.2·10^−17 ^cm (pyrene), 1.2·10^−17 ^cm (anthracene), 0.8·10^−17 ^cm (naphthalene), 2.0·10^−17^ (xylene), 1.2·10^−17 ^cm (toluene), and 0.7·10^−17 ^cm (benzene). Values reported in the EPA database for pyrene, anthracene, and naphthalene are 1.7·10^−17 ^cm, 2.3·10^−17 ^cm, and 1.2·10^−17 ^cm, respectively.^22^ In addition, Etzkorn et al. have presented values of 2.5·10^−17 ^cm, 1.9·10^−17 ^cm, and 1.0·10^−17 ^cm for xylene, toluene, and benzene, respectively.^24^ The experimental results are lower, but the values from the literature have been determined from the entire C–H region, starting at wavenumber 2700 cm^−1^, whereas the experimental results were evaluated for the wavenumber interval given above. The difference in spectral range could, to some extent, account for the lower values. Nevertheless, the results can be utilized for calibration, and naphthalene was selected to represent tars in the evaluation of data from the gasifier due to its prevalence in biomass tars.^1,7^ The range of slopes and integrated absorption cross-sections for the investigated compounds suggest that the choice of naphthalene to represent all tars introduces an uncertainty of up to 30–40% of the evaluated tar concentrations.

### FT-IR Gasification Measurements Using Open-Ended Cell

**Figure 3.**
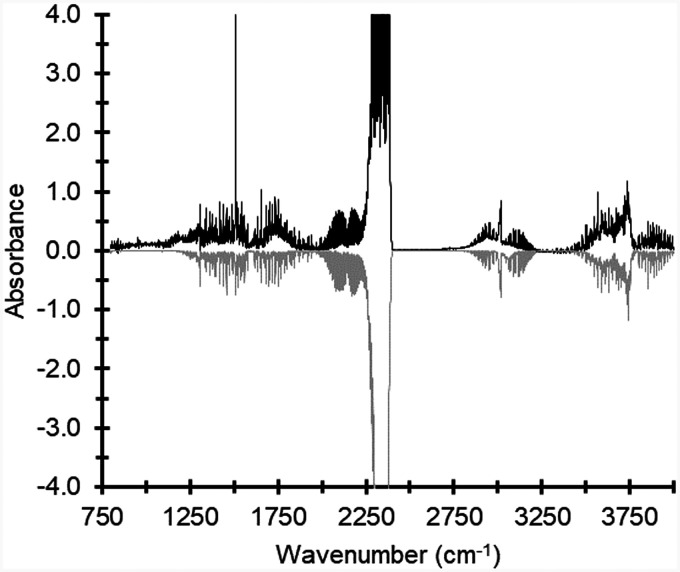
FT-IR spectrum measured during gasification of wood pellets (black) and spectrum simulated using the HITRAN database and tar calibration spectra (gray).

Figure 3 shows an FT-IR spectrum with peaks of CO, CO_2_, CH_4_, and H_2_O, measured during the gasification of wood pellets. Also, a signature of oxygenated hydrocarbons is observed at wavenumbers around 1750 cm^−1^.

Spectral lines of methane are located at wavenumbers 2700–3000 cm^−1^ and positioned on top of a broad spectral signature originating from other hydrocarbons, such as tars. The spectrum plotted with negative absorbance was obtained from a fit of computed spectra to the experimental spectrum. The calculated spectra were based on absorption data from the HITRAN^20,21^ database and the tar naphthalene calibration measurements. The assigned tar component included wavenumbers above 3020 cm^−1^ (cf. Fig. 2a) and the fitted spectrum only reproduces the spectral signature located above wavenumber 3020 cm^−1^.

**Figure 4.**
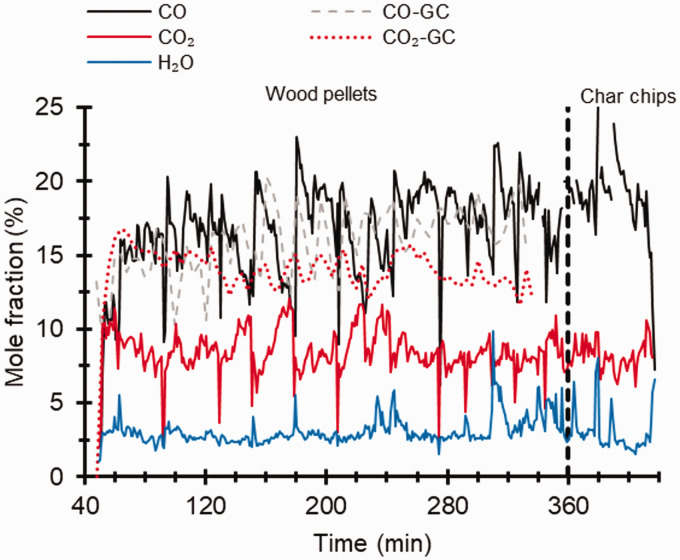
Time profiles of CO, CO_2_, and H_2_O evaluated from FT-IR spectra and measured using micro-GC during gasification of wood pellets (0–360 min) and charcoal (360–420 min).

Figure 4 shows the evaluated concentrations of CO, CO_2,_ and water, presented as mole fractions, from the gasifier for about five hours of operation using wood pellets and char chips. About 50 min after the start-up, raw gas from the gasifier was redirected through the hot cell, which can be seen by a rapid increase in measured concentrations at this time (cf. Fig. 4). While gas concentrations during wood pellet operation (0–360 min) were rather stable, a gradual reduction in CO was observed during gasification of char chips (360–420 min), mainly due to the lower presence of volatiles in the biomass char.

Mole fractions of CO evaluated from FT-IR reside around 15% and show good agreement with values obtained using GC. An accuracy of concentrations of CO, CO_2_, and CH_4_ evaluated from FT-IR spectra has been determined by calibration measurements on a gas mixture, and the results are presented in Figure S1 (Supplemental Material). The error for CO has been determined to 2% at these concentration levels. For CO_2_, concentrations measured by GC as well as readings of the IR instrument (cf. Fig. 4 and Figure S2) are higher than values obtained from FT-IR. The absorption of CO_2_ is strong in the infrared regime; in Fig. 4, it can be seen that the major part of the CO_2_ spectrum shows a saturated signal where essentially no light is transmitted to the detector. Due to the strong absorption, data evaluation was made at the left flank of the CO_2_ spectrum. While measurements on the calibration gas show that the CO_2_ concentration was overestimated, by ∼2% at 10% concentration, comparison with the GC and IR results indicates that the results evaluated from the noisier FT-IR data measured in the gasifier are underestimated. Improved accuracy for major species with strong absorption can be obtained by adjusting the gas dilution. However, this also reduces signals from minor components of interest such as tar compounds and needs to be considered when selecting operating conditions for the online FT-IR measurement.

Steep changes observed in CO and CO_2_ values represent intentional operational disturbances. The bed was stirred momentarily such that the pellet density of the bed decreased and created less resistive paths through which gases with higher tar concentrations could pass. Thus, the online FT-IR measurement readily captures instantaneous changes in gas composition. During such intermittent disturbances, the gas flow into the heated cell increased marginally; however, data are not corrected for these fluctuations in the flow. As the focus was to prove the concept of online FT-IR measurements on the raw gas using the heated cell, a detailed interpretation of the specific gasification process was beyond the scope of this study.

**Figure 5.**
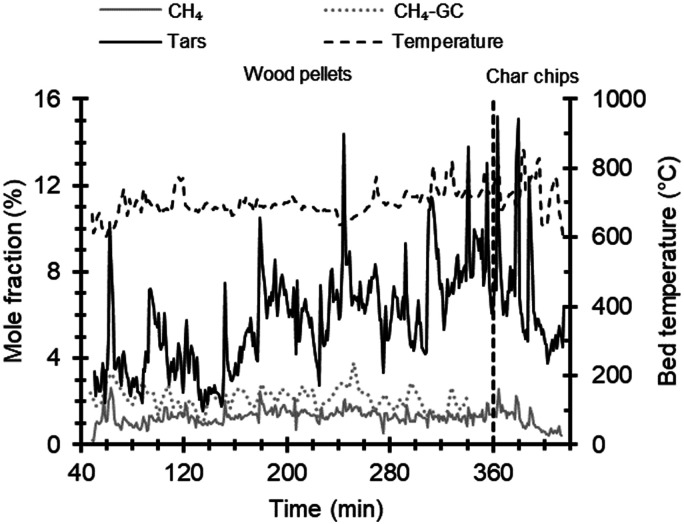
Time profiles of concentrations for CH_4_, measured using FT-IR and GC, and aromatic tars, measured using FT-IR, during gasification of wood pellets (0–360 min) and char chips (360–420 min) with varying bed temperature. Tars denote the concentration obtained by an evaluation based on naphthalene, as described in the Results section. The evaluated tar concentrations have been multiplied by a factor of 10 in the graph to facilitate comparison with the CH_4_ profile.

Figure 5 shows time profiles for methane and tars, as well as bed temperature during the gasification of wood pellets and char chips. Marginal changes in the tar concentrations during the gasification of wood pellets are expected as the bed temperature was maintained at 700 ± 50 ℃. Though the bed temperature was reduced gradually after about 360 min of operation, reductions in tars and CH_4_ are attributed to the addition of char chips resulting in reduced release of volatiles during gasification. Tar concentrations of around 0.4% would correspond to 10 g/m^3^, which is in good agreement with values for downdraft gasifiers compiled by Milne et al.^1^ Thus, with appropriate choice of reference species and spectra for data evaluation, reasonable quantitative tar concentrations can be retrieved from FT-IR measurements.

**Figure 6.**
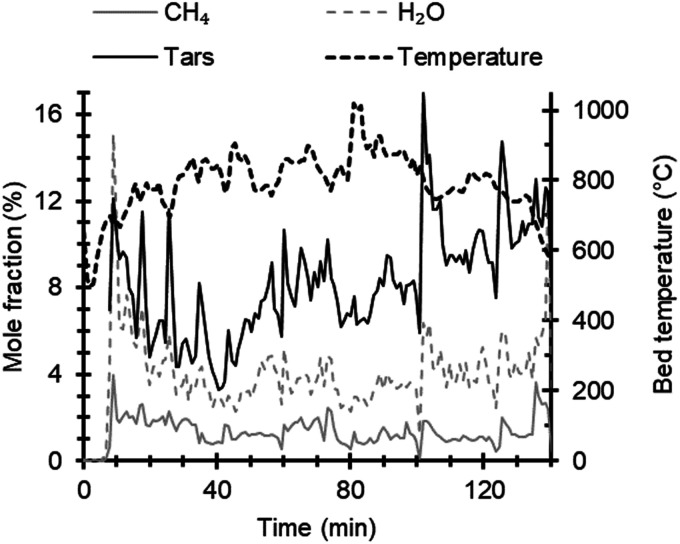
Time profiles for CH_4_, H_2_O, tars, and bed temperature using bark pellets. Tars denote the concentration obtained by an evaluation based on naphthalene, as described in the Results section. The evaluated tar concentrations have been multiplied by a factor of 10 in the graph to facilitate comparison with the other data.

Figure 6 shows time profiles for methane, water, tars, and bed temperature during the gasification of bark pellets and demonstrates the ability to follow operational changes in the FT-IR data. A decrease in the concentrations of tar and methane can be seen while the bed temperature was increased gradually from about 700 ℃ to 1000 ℃. The opposite trend is observed when the temperature decreases after ∼100 min. The bed temperature was controlled by adjusting the airflow for the gasification, which could facilitate or suppress the oxidation of gases, tars, and char, respectively. This would also increase or reduce the temperature in the oxidation zone. Consequently, tar concentrations are expected to reduce or increase due to the changes in oxidation and thermal cracking. Reducing and increasing trends in the tar concentrations are in line with the results presented by Milne et al.^1^

**Figure 7.**
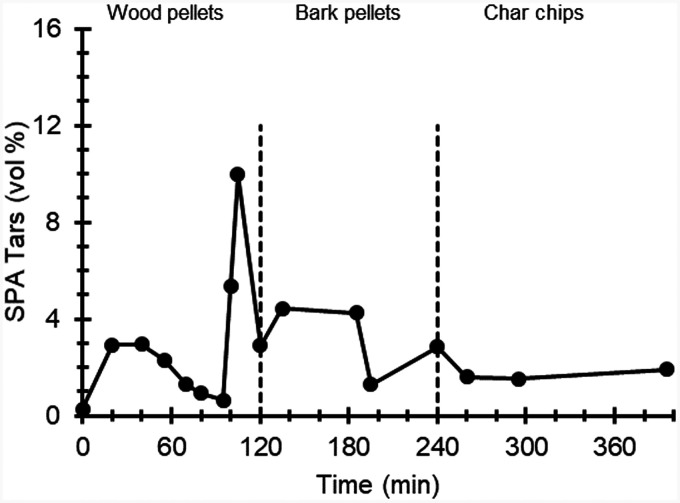
Tar concentrations measured using solid-phase adsorption (SPA) while the gasifier was operated with wood pellets (0–120 min), bark pellets (120–240 min), and char chips (240–400 min). Measured tar concentrations have been multiplied by a factor of 10 in the graph to facilitate comparison with FT-IR data in Figs. 5 and 6.

Figure 7 shows tar concentrations obtained from SPA measurements while the gasifier was operated on wood pellets (0–120 min), bark pellets (120–240 min), and char chips (240–400 min). Gravimetric values of tars obtained using the SPA technique were in the range 0.7–25 g/m^3^, 25 ℃, and converted into volume percentage using the ideal gas law and considering all tars to be naphthalene. For consistency with Fig. 6, SPA tar concentrations were also multiplied by a factor of 10 for presentation in Fig. 7. The SPA values are in close agreement with the FT-IR tar concentration levels, as shown in Fig. 6, which as mentioned previously show good agreement with values presented by Milne et al.^1^ Comparing FT-IR and SPA results, it should be noted that the tar concentration varies during the process, as shown in the FT-IR data, measured at a higher acquisition rate, and the value obtained at a specific point in time depends on when fresh fuel was added. The addition of new fuel can spontaneously increase the concentration of tars due to the release of volatiles and tars in excess, which is then gradually reduced as the fuel is converted into char. During operation, fresh fuel was added to maintain the product gas formation downstream of the gasifier. The specific times for the addition of fuel were different for the runs with FT-IR measurements and SPA sampling, but the amount of fresh fuel added each time and the bed temperature was similar.

In addition to the open-ended cell, measurements were also conducted with the cell closed using KBr windows. This was done to fulfill safety norms for field measurements and safer design of the ventilation system for the product gas downstream of the heated cell. The closed cell was operated for a couple of hours (Figures S3 and S4, Supplemental Material), and the concentrations of permanent gases and tars are in agreement with results obtained for the open-ended cell (cf. Figs. 6 and 7) within the experimental uncertainty. Measurements in the open-ended cell could be more sensitive to external disturbances, and CO data for the closed cell indicate less variation. Less disturbance is definitely an advantage for a configuration with a closed cell. However, care must be taken to avoid condensation on windows; for example, a thin layer of condensed tars was observed on the KBr windows after measurements. It was not clear if this tar was accumulated during gasifier operation or formed due to condensation after completion of the measurements when the cell was no longer flushed with nitrogen. The formation of window deposits needs to be investigated in the future with longer trial runs and the development of a suitable system to minimize the deposition of tars on the windows.

## Conclusion

The main conclusions of this study can be summarized as follows: An external heated cell connected to an FT-IR instrument allowed for online measurements in a harsh gasification environment. Instant changes in the composition of permanent gases and water could clearly be monitored. Moreover, online signatures of tars could be obtained, and data correlated well with the widely practiced SPA technique and operational changes in a bench-scale downdraft gasifier. If the emphasis is not to measure specific tar species, data obtained with online FT-IR measurements could be valuable input for process control. The heated cell arrangement can thus be applied for online FT-IR field measurements.

## Supplemental Material

sj-pdf-1-asp-10.1177_0003702821991891 - Supplemental material for Infrared Spectroscopy for Online Measurement of Tars, Water, and Permanent Gases in Biomass Gasification Click here for additional data file. Supplemental material, sj-pdf-1-asp-10.1177_0003702821991891 for Infrared Spectroscopy for Online Measurement of Tars, Water, and Permanent Gases in Biomass Gasification by Mohit Pushp, Christian Brackmann and Kent Davidsson in Applied Spectroscopy

## Supplemental material

All supplemental material mentioned in the text, consisting of Figures S1 to S4 and Table S1, is available in the online version of the journal.
