# Supplementary material for: Infrared Spectroscopy for Online Measurement of Tars, Water, and Permanent Gases in Biomass Gasification
Source: Appl Spectrosc. 2021 Feb 18;75(6):690–7. doi: 10.1177/0003702821991891 (PMC8209763; doi:10.1177/0003702821991891)
Supplement: sj-pdf-1-asp-10.1177_0003702821991891 - Supplemental material for Infrared Spectroscopy for Online Measurement of Tars, Water, and Permanent Gases in Biomass Gasification [file sj-pdf-1-asp-10.1177_0003702821991891.pdf]

Supplementary Material

Infrared spectroscopy for online measurement of tars, water, and permanent gases in biomass gasification

[1,2]M. Pushp, [3]C. Brackmann, [1]K. Davidsson

[1]Research Institutes of Sweden, Borås, Sweden.
[2]Department of Chemistry and Molecular Biology, University of Gothenburg, Sweden.
[3]Division of Combustion Physics, Lund University, Lund, Sweden.

FT-IR calibration of permanent gases

Calibration was made in the closed cell with KBr windows mounted, at atmospheric pressure and the cell temperature set to 400 °C with a mix of gases, specified in Table 1, that resembles the product gas from a biomass gasifier.

Table S1. Concentrations of permanent gases used for calibration. The uncertainties of ±2% are relative to the specified molar fractions.

Permanent gas	Molar fraction (%)
CO	29.9 ± 2%
CO_2	10.0 ±2%
CH_4	10.01 ± 2%
H_2	39.9 ± 2%
N_2	4.06 ± 2%
$C_3H_8 + C_2H_2 + C_2H_4 + C_2H_6$	0.5 + 0.491 + 1.01 + 3.99 = 5.991±2%

Measurements at different concentrations were made by dilution of the gas mix with nitrogen. Results from analyses of CO, CO_2, and CH_4 are presented in Figure S1a, where the evaluated mole fractions are plotted versus the set mole fractions of the mixes. The dashed line represents points where evaluated and set concentrations would be equal. Up to mole fractions of 10 % for CO and 5% for CO_2 and CH_4, the absolute error in the evaluated concentration is less than 1%. While the error for CH_4 remains below 1% up to a mole fraction of 10%, the error for CO and CO_2 increases. At the maximum mole fractions for CO and CO_2, 29.9 and 10.0%, the error is 3 and 2%, respectively. The increasing error may be due to partially saturated absorption for which the Beer-Lambert law is no longer directly valid. The uncertainties of the calibration

mixture given in Table S1 represent percentages of the specified fractions. Uncertainties for the set mole fractions in the experiments have been estimated from the values of Table S1 combined with uncertainties in mass flow controller readings assumed to be 1% of full scale. The uncertainties in set mole fractions are thus estimated to be 0.2-0.7% for CO and 0.1-0.2% for CO_2 and CH_4. Figure S1b-d show measured and fitted spectra of CO, CO_2, and CH_4 at the lowest mole fractions with good agreement between experimental data and simulations.

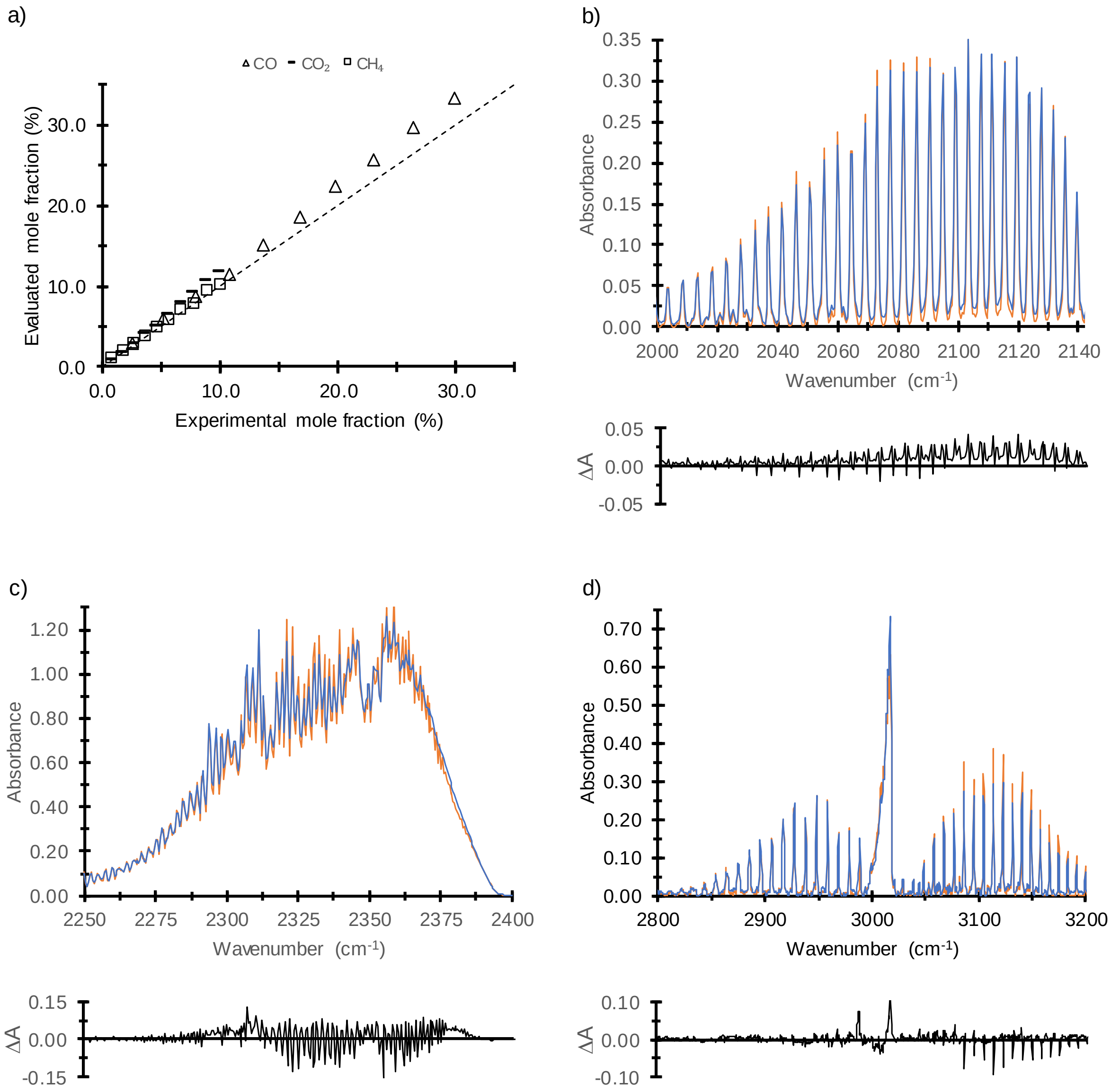

Figure S1. Calibration of the FT-IR setup with the external cell at temperature 400 °C using a mixture of permanent gases CO, CO_2, and CH_4 with known concentrations at levels similar to the product gas from gasification. a) Graph of evaluated concentrations versus mixture composition. Experimental FT-IR spectra (blue) and fitted HITRAN spectra (red) spectra for calibration gas b) CO (2.9%), c) CO_2 (0.9%), and d) CH_4 (1.0%). Residual plots below each graph shows the difference (ΔA) between the experimental and the fitted spectrum.

Comparison with IR instrument

Figure S2 shows concentrations of CO_2 and CH_4 obtained during gasification from the separate infrared instrument and FT-IR measurements. Sharp drops in the CO_2 concentration obtained from the FT-IR appear because the cell is directly connected to the gasifier, and intermittent disturbances in gasifier operation due to the stirring of the bed are captured.

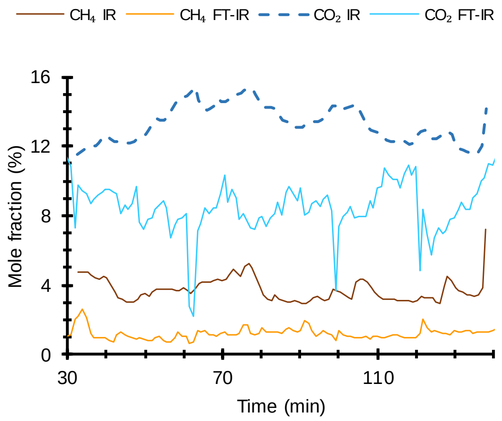

Figure S2. Comparison of CH_4 and CO_2 concentrations obtained from the IR (dry gas) and FT-IR instruments (wet gas) measured during the gasification of wood pellets.

Mole fractions of CO_2 and CH_4 measured by the IR instrument are higher than results obtained with FT-IR. In contrast to FT-IR, which measures the wet gas, the IR instrument measured the dry gas, which had been cooled to 20 °C. Measurements on dry gas result in marginally higher readings for the IR instrument compared with FT-IR and does probably not fully account for the observed differences. The gas monitored by the IR device was cleaned using a particle filter to avoid damage to the instrument, whereas the particle-laden raw gas was passed through the heated cell. The presence of particles may affect the FT-IR measurements via extinction, i.e., absorption and scattering of light by the particles. However, extinction should mainly affect the spectrum baseline, which is compensated for in the data evaluation. Nevertheless, uncertainties in baseline compensation and some impact of extinction cannot be ruled out completely. However, the comparison suggests that evaluated FT-IR data from the gasification could be underestimated.

Measurements with closed cell

Figure S3 and S4 present results obtained during gasifier operation with a closed cell for the FT-IR measurements.

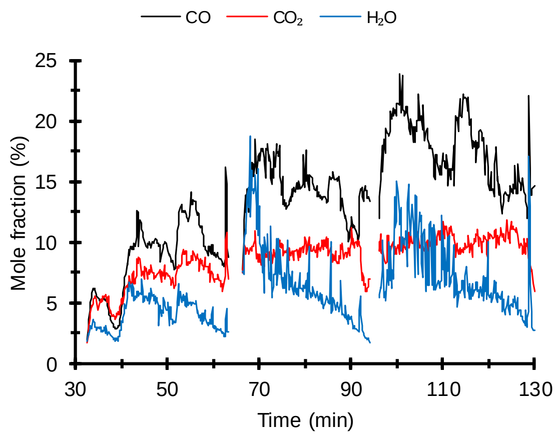

Figure S3. Time profiles of permanent gases and water measured using a closed cell, while the gasifier was operated with wood pellets. Fresh fuel was added between 60 – 70 min and 90 – 100 min. Data with disturbances, measured during the manual fuel feeding, have been removed intentionally for a clearer presentation of the stable gasifier operation.

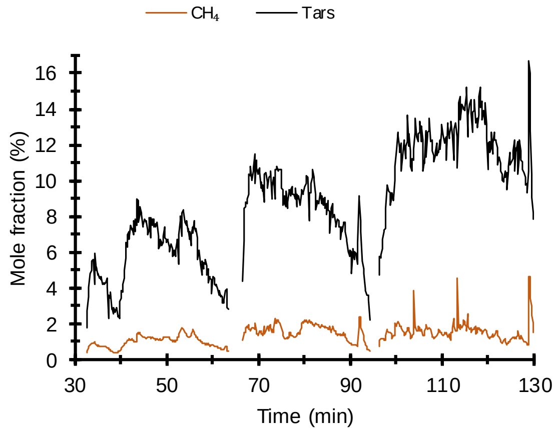

Figure S4. Time profiles of methane and tars measured using closed cell, while the gasifier was operated using wood pellets. Fresh fuel was added between 60 – 70 min and 90 – 100 min. Data with disturbances, measured during the manual fuel feeding, have been removed intentionally for a clearer presentation of the stable gasifier operation. The evaluated tar concentrations have been multiplied by a factor of 10 in the graph to facilitate comparison with the CH_4 data.

In the arrangement with a closed cell, the lab-scale gasifier was operated in a batch mode with fresh fuel added between 60 – 70 min. and 90 – 100 min of operation time. Data acquired at the

time of addition of fuel has been removed intentionally from Figure S4 and S5, as the gas flow was disturbed. Increased concentrations in CO, CH_4, H_2O, and tars can be seen after the addition of fresh fuel, which then comes in contact with the heated bed and undergoes devolatilisation. Concentrations of the species mentioned above later reduce with time as the fuel dried and was converted into char.
